# Factors associated with risky sexual behaviour among sexually experienced undergraduates in Osun state, Nigeria

**DOI:** 10.4314/ahs.v22i1.6

**Published:** 2022-03

**Authors:** Akinlolu Omisore, Ifeoluwa Oyerinde, Omoniyi Abiodun, Zainab Aderemi, Titilayo Adewusi, Iseoluwa Ajayi, Temitope Fagbolade, Sukurat Miskilu

**Affiliations:** 1 Community Medicine; 2 Osun State University College of Health Sciences, Community Medicine

**Keywords:** Behaviour, experienced, factors, risky, sexual undergraduates

## Abstract

**Background:**

Risky sexual behavior (RSB) is common among youths which predispose them to sexually transmitted infections. This study sets out to identify the factors associated with RSB among undergraduates in Osun state.

**Methods:**

The study design was descriptive cross sectional and a total of 550 respondents from two universities in Osun state were sampled using a multistage sampling technique, out of which data from 266 sexually experienced respondents was further analyzed. Data collected via a semi-structured self-administered questionnaire and analyzed using univariate, bivariate and multivariate analyses.

**Results:**

The 266 respondents consisted of 54.5% males and 45.5% females and larger percentage of them were in the age group 15–24years. Seven out of ten respondents (69.9%) were sexually active while 65.8% were involved in at least one RSB. Of the 266 respondents, 28.6% ever had concurrent multiple sexual partners, 15.8% used alcohol/drugs at last sex while 48.1% did not use condom at last sex. More males (71.7%), alcohol users (76.8%), drug users (78.0%), pornography watchers (82.7%), internet users (71.2%) respondents “not in good terms with mum” (86.7%) and “those whose mum doesn't instruct them morally” (84.2%) were involved in RSB compared to their respective counterparts (p<0.05). However, there were no identifiable predictors of RSB on regression analysis.

**Conclusion:**

Risky Sexual Behavior is prevalent among undergraduates with males being more involved, among other associated factors. Concerned stakeholders should engage youths via behavioral change communication strategies so as to significantly reduce their involvement in RSB.

## Introduction

Risky sexual behaviours (RSB) are defined as behaviors leading to health vulnerabilities such as sexually transmitted infections (STIs) and unintended pregnancies. These RSB include having multiple sexual partners, inconsistent use of condom, and early sexual debut^1^. Some authors have added to the list of RSB: sexual activity done under the influence of alcohol, anal intercourse, sexual violence and transactional sex^2^, intravenous drug abuse^3^, sexual orientation anduntreated sexually transmitted diseases^4^. Many of these behaviours are very common among youths and young adults due to the ‘general risk taking’ behaviour and unusual overconfidence of members of this age group^5^,^6^. Young people constitutes about a fifth of the world's population and in many developing countries are a far more significant proportion of the population. Majority of sexually active persons are youths aged 15–24, it is the age group in which exploration of sexuality and sexual relationship begin making them accountable for about one-half of all new sexually transmitted infections due to their susceptibility^7^. These foundational years offer an ideal period of opportunity for building the fundamentals of sexual and reproductive health and rights among young people and for preparing them to avoid risky sexual behaviors^7^.

Nigeria with an estimated population of 200 million has the second largest HIV epidemic in the world and one of the highest rates of new infections in sub-Saharan Africa^8^. Out of 1.9million people living with HIV in 2018, about 1.5% were within age 15–49 years although efforts have been made to limit the spread of HIV/AIDS in the country but it still retains an upward trajectory in certain states due to engagement of youths in risky sexual behaviours^4^ which is as a result of their curiosity, enthusiasm and keenness in attempting various new things neglecting the negative consequences of such deeds^5^. It was reported in a study on risky sexual behaviour of young people in an urban Nigerian community that lack of knowledge about HIV/AIDS, other STIs and poverty are factors that increase the chances of young people engaging in RSB^7^.

Involvement/ engagement of undergraduate youths in RSB cannot be ruled out because larger proportion of this bracket are single, young, hyperactive in nature and get excited with the unlimited freedom of staying away from their parents/ liberal conditions of the campus. They also believe they have the sovereignty to handle their own affairs and take certain decisions which predisposes them to highly risky sexual activities^5^. Thus, the prevalence of RSB among the youths is overwhelming and its complications/consequences still remain the major health problems of youths and young adults worldwide. Hence, this study sets out to assess the prevalence of RSB and identify the possible factors or determinants that are associated with it among undergraduates.

## Materials and methods

### Study design

This descriptive cross sectional study was carried out in June 2019 among undergraduates of tertiary institutions in Osun state, South West Nigeria.

### Sample size determination

The sample size was estimated using Leslie Fischer's formula for single proportion (more than 10,000) and a prevalence rate of 47.4% from a previous study^9^ was used to estimate the sample size of 380 which was increased to 550 to account for attrition and non-responses and as well allow for adequate cross tabulations and multivariate analysis. Out of the 550 respondents, 266 were sexually experienced, that is those who ever had sexual intercourse and were considered eligible for further analyses. This is in order to appropriately compare those involved in risky sexual behaviours with those who are sexually experienced but are not involved in risky behaviours.

### Study Population and Sampling technique

A multistage sampling technique was used in identifying the sample for the study. At the first stage, two universities (Osun State University and Obafemi Awolowo University, Ile-Ife) were selected through simple random sampling via balloting among the seven active tertiary institutions situated in Osun state. At the second stage, two colleges/faculties were taken from each of the two universities via same process. During the third stage, four departments were chosen from each of the selected faculties by simple random sampling via balloting. At the fourth stage, proportionate sampling was used to determine the number of respondents to be taken from eachdepartment based on the total sample size required and the population of final year students in each department. Thereafter some of the students present in class on the day of data selection were selected via systematic random sampling. The inclusion criterion was for the respondent to be a final year student on account of allowing adequate time for exposure to RSB.

### Research Instrument

The questionnaire was pretested in a private owned university located at Ede and was validated. The questionnaire comprised three sections. Section A focused on respondents' socio-demographic variables, Section B focused on respondents' sexual behaviour and Section C focused on likely factors associated with risky behaviour. The questionnaire was administered by the authors after sessions of training by the lead author, the questionnaire was self-completed/administered after the purpose of the study was explained to the respondent. A semi-structured questionnaire was distributed to the selected students for self- administration after the purpose of the study had been explained to them in details.

### Ethical considerations

Ethical approval/ clearance was sought and obtained from the Health Research and Ethics Committee, College of Health Sciences, Osun State University. Permission was taken from the Head of Departments of selected departments. Confidentiality of information and respondents identities were assured and maintained throughout the study.

### Data management

The data obtained was analyzed and results were presented using relevant frequency distribution tables and charts, chi-square and logistic regression analyses were generated with confidence level set at 95% and a p-value < 0.05 considered as significant. It was only variables that were significant in the bivariate analysis (chi-square) that were added to the regression models.

### Measures

In this study those that answered “yes” to the variable “have you ever had sexual intercourse” were regarded as sexually experienced and those that “have had sex in the past 12 months/ one year” were referred to as sexually active. Risky sexual behaviour is defined as any sexually experienced person who has been involved in at least one of; inconsistent use of condom (did not use it at all or 100% of the time), use of alcohol/ drugs before sex and having multiple sexual partners concurrently. Those involved in any of these behaviours were scored “1” while those uninvolved were scored “0” and the three variables were added together. Those that scored 1 and above were classified to be involved in RSB and those that scored 0 were not involved in RSB.

## Results

Table 1 shows the socio-demographic characteristics of the 266 sexually experienced respondents. The proportion of male respondents was slightly higher (54.5%) than females (45.5%). The mean age was 22.92±2.28 years with 74.4% of the respondents in the age group 15–24 years while a quarter were aged 25–25 years.

**Table 1:** Socio-demographic characteristics sexually of experienced respondents

VARIABLES	FREQUENCY (n=266)	PERCENTAGE (%)
**Sex**		
Male	145	54.5
Female	121	45.5
**Age –group** (Mean=22.92, SD =2.28, Min =18, Max= 35#)		
Youths (15–24)	198	74.4
Young adults (25–35)	68	25.6
**Religion**		
Christianity	179	67.3
Islam	81	30.5
Traditional	6	2.2
**Ethnicity**		
Yoruba	237	89.1
Others (Igbo, Niger delta)	29	10.9
**Mother's highest education attained**		
None	7	2.6
Primary	17	6.4
secondary	77	29.0
Tertiary	165	62.0
**Father's highest education attained**		
None	9	3.4
Primary	13	4.9
Secondary	63	23.7
Tertiary	181	68.0
**Parental marital status**		
Currently married	222	83.5
Not currently married	44	16.5
**Type of family**		
Monogamous	202	75.9
Polygamous	64	24.1
**Average monthly income (minimum wage =18000)**		
Below minimum wage	204	76.7
Minimum wage and above	62	23.3

**Figure 1:**
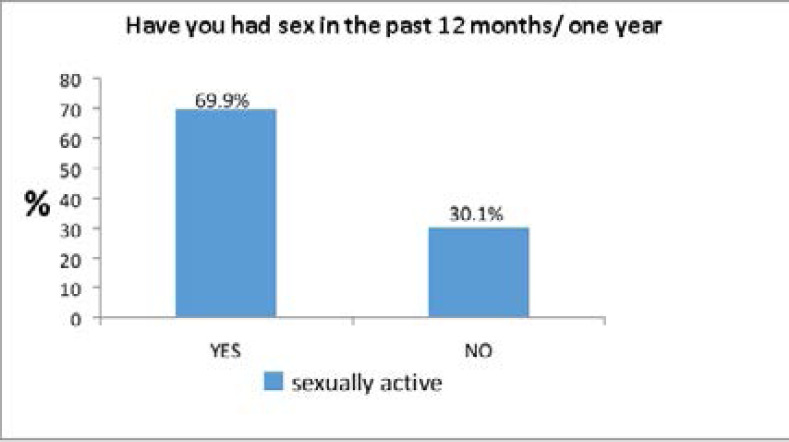
proportion of respondents who are sexually active (n=266)

Figure 1 shows that out of the 266 sexually experienced respondents 69.9% (186) were sexually active while 30.1% (80) were not sexually active.

Table 2 shows the sexual and dating characteristics of respondents who were sexually experienced by gender. The mean age at first sexual intercourse was 18.11±3.48 and the median age was 18.00, almost half (46.3%) of them had their sexual debut at age 15–19 followed by 27.4 % who had their first sexual intercourse between age 20–23 years while 8.2% were unable to remember their age at first sexual intercourse as at the time the data were collected. at the various age groups Except for the age group 15–19 years, there were more males than females who had their sexual debut at each of the different age groups. The percentage of respondents that have had sexual intercourse in the last one year (sexually active respondents) was 69.9%) while 42.5% have had sexual intercourse within months, 16.1% had sexual intercourse within weeks and 11.3% had sexual intercourse within days. Less than one-third of the respondents (28.6%) have had more than one sexual partner concurrently.

**Table 2:** Respondents' sexual and dating characteristics by gender

Variables	Sub-categories of variables	Sex		Total N=266 (%)	Remarks
		Male 145(%)	Female 121(%)		
**Ever engaged in the use of internet for** **sexual purposes**	Yes	103(63.6)	59 (36.4)	162(60.9)	^2^ =13.743 p<0.001*
	No	42 (40.4)	62 (59.6)	104(39.1)	
**Age at first sexual intercourse** (Mean= 18.11, SD= 3.480, Median= 18.00, Min= 10, Max=27)	10–14 years	20 (55.6)	16 (44.4)	36 (13.5)	^2^ =2.784 p =0.585
	15–19 years	61 (49.6)	62 (50.4)	123(46.3)	
	20–23 years	45(61.6)	28 (38.4)	73 (27.4)	
	24–27 years	7 (58.3)	5 (41.7)	12 (4.5)	
	Can't remember	12 (54.5)	10 (45.5)	22(8.3)	
**The last time respondent had sexual** **intercourse**	Days ago	19 (63.3)	11 (36.7)	30(11.3)	^2^ =2.620 p =0.454
	Weeks ago	22 (51.2)	21 (48.8)	43(16.1)	
	Months/A year ago	65 (57.5)	48 (42.5)	113(42.5)	
	Years ago	39 (48.8)	41 (51.2)	80(30.1)	
**Respondent ever had more than one sexual** **partner concurrently**	Yes	54 (71.1)	22 (28.9)	76(28.6)	^2^=11.741 p=0.001*
	No	91 (47.9)	99 (52.1)	190(71.4)	
**Respondent ever been in a dating or** **courtship relationship**	Yes	126(54.3)	106(45.7)	232(87.2)	^2^ =0.030 p =0.864
	No	19 (55.9)	15 (44.1)	34(12.8)	
**Respondent currently in a dating or** **courtship relationship (n=232)**	Yes	78 (48.1)	84 (51.9)	162(69.8)	^2^ =8.216 p=0.004*
	No	48 (68.6)	22 (31.4)	70(30.2)	
**How long is this current relationship** **(n=162)**	Less than 6 months	57 (49.1)	59 (50.9)	116(71.6)	^2^ =0.160 p =0.689
	6 months and above	21 (45.7)	25 (54.3)	46(28.4)	
**Respondent ever been treated of any** **sexually transmitted disease**	Yes	14 (45.2)	17 (54.8)	31(11.7)	^2^ =1.237 p =0.266
	No	131(55.7)	104(44.3)	235(88.3)	
**Number of sexual partner respondent ever** **had**	One partner	39 (39.8)	59 (60.2)	98(36.8)	^2^ =13.550 p<0.001*
	Two or more partners	106(63.1)	62 (36.9)	168(63.2)	
**Condom usage at last sexual intercourse**	Yes	77 (55.8)	61 (44.2)	138(51.9)	^2^ =0.191 p =0.662
	No	68 (53.1)	60 (46.9)	128(48.1)	
**Use of condom at all sexual activities (100%** **of the time)**	Yes	69 (56.1)	54 (43.9)	123(46.2)	^2^ =0.232 p =0.630
	No	76 (53.1)	67 (46.9)	143(53.8)	
**Alcohol intake / use of drug for sexual** **activity at last sexual intercourse**	Yes	28 (66.7)	14 (33.3)	42(15.8)	^2^ =2.972 p =0.085
	No	117(52.2)	107(47.8)	224(84.2)	
**Ever forcefully had sex with anyone**	Yes	20 (57.1)	15 (42.9)	35(13.2)	^2^ =0.113 p =0.737
	No	125(54.1)	106(45.9)	231(86.8)	
**Respondent ever raped by someone**	Yes	18 (47.4)	20 (52.6)	38(14.3)	^2^ =0.912 p =0.340
	No	127(55.7)	101(44.3)	228(85.7)	

The proportion of respondents that have ever had only “one sexual partner” thus far in life was 36.8% while those that have had two or more sexual partners i.e. multiple partners whether at the same or at different times was 63.2%. About a tenth (11.7%) of those that were sexually experienced said they have been treated of sexually transmitted disease before. Among the sexually experienced, those that used condom at last sexual intercourse was 51.9% while consistent use of condom i.e. 100% all the time among the sexually experienced was 46.2%. Majority of the respondents (84.2%) did not take alcohol or use drug for sexual activity at last sexual intercourse. 13.2% said they ever forcefully had sex with anyone and 14.35% stated someone has forcefully had sex with them. There were statistically significant differences between males and females with respect to the variables “Ever engaged in the use of internet for sexual purposes”; “Ever had more than one sexual partner concurrently”; “Currently in a dating or courtship relationship”; and “Number of sexual partners ever had”. More males engaged in the use of internet for sexual purposes (63.6%), had more than one sexual partner concurrently (71.1%) and have also had two or more sexual partners (63.1%) in their lifetime while more females (51.9%) are currently in a dating or courtship relationship.

Table 3 shows the relationship between respondents' involvement in RSB and their socio-demographic characteristics. There is no statistically significant difference regarding involvement in risky sexual behavior and the variables age, religion, ethnicity, mother's highest education, father's highest education, parental marital status, type of family and average monthly income as the p value was more than 0.05. Over two third of the male respondents were involved in RSB (71.7%) compared to less than three fifths of females (58.7%) at p= 0.026

**Table 3:** The relationship between respondents' involvement in Risky Sexual Behavior and their socio-demographic characteristics

VARIABLES	SUB VARIABLES	Not INVOLVED IN RSB (%)	INVOLVED IN RSB (%)	REMARKS
Sex	Male	41 (28.3)	104(71.7)	^2^ = 4.988 p = 0.026*
	Female	50 (41.3)	71 (58.7)	
Age	Youths (15–24)	65 (32.8)	133 (67.2)	^2^ = 0.657 p = 0.417
	Young adults (25–35)	26 (38.2)	42 (61.8)	
Religion	Christianity	65 (36.3)	114 (63.7)	^2^ = 1.109 p = 0.570
	Islam	24 (29.6)	57 (70.4)	
	Traditional	2 (33.3)	4 (66.7)	
Ethnicity	Yoruba	79 (33.3)	158 (66.7)	^2^ = 0.743 p = 0.389
	Others	12 (41.4)	17 (58.6)	
Mother's education	Lower education	9 (37.5)	15 (62.5)	^2^ = 0.127 p = 0.722
	Higher education	82 (33.9)	160 (66.1)	
Father's education	Lower education	10 (45.5)	12 (54.5)	^2^ = 1.347 p = 0.246
	Higher education	81 (33.2)	163 (66.8)	
Parental marital status	Currently married	73 (32.9)	149 (67.1)	^2^ = 1.051 p = 0.305
	Not currently married	18 (40.9)	26 (59.1)	
Type of family	Monogamous	72 (35.6)	130 (64.4)	^2^ = 0.766 p = 0.381
	Polygamous	19 (29.7)	45 (70.3)	
Average monthly income (minimum wage=#18,000)	Below minimum wage	74 (36.3)	130 (63.7)	^2^ = 1.657 p = 0.198
	Minimum wage and above	17 (27.4)	45 (72.6)	

#**Lower education**: no formal education and primary education

#**Higher education**: secondary and tertiary education

Table 4 outlines some factors that associated with involvement in Risky Sexual Behavior among the respondents that are sexually experienced. The respondents that “have ever been treated of any sexually transmitted disease” were more involved in RSB (83.9%) compared to those who “have not been treated of any STD (63.4%) with p = 0.024. Those that ever had two or more sexual partner were more involved in RSB (71.4%) compared to those that had one sexual partner at p = 0.011

**Table 4:** Selected personal, family and social factors and their association with RSB

VARIABLES	SUB VARIABLES	Not INVOLVED IN RSB (%)	INVOLVED IN RSB (%)	REMARKS
Are you currently in a dating/ courtship	Yes	52 (30.1)	121 (69.9)	^2^ = 3.791 p = 0.052
	No	39 (41.9)	54 (58.1)	
Have you ever been treated of any STI?	Yes	5 (16.1)	26 (83.9)	^2^ = 5.097 p = 0.024*
	No	86 (36.6)	149 (63.4)	
Number of sexual partner ever had	One partner	43 (43.9)	55 (56.1)	^2^ = 6.443 p = 0.011*
	Two or more	48 (28.6)	120 (71.4)	
Have you ever forcefully had sex with anyone	Yes	7 (20.0)	28(80.0)	^2^ = 3.908# **p = 0.048#**
	No	84 (36.4)	147 (63.6)	
Has someone ever forcefully had sex with you	Yes	16 (42.1)	22 (57.9)	^2^ = 1.228 p =0.268
	No	75 (32.9)	153 (67.1)	
Are you in good terms with Mum?	Yes	87 (36.9)	149 (63.1)	^2^ = 6.548 p = 0.010*
	No	4 (13.3)	26 (86.7)	
My Mum instruct me morally	Yes	85 (37.3)	143 (62.7)	^2^ = 6.684 p = 0.010*
	No	6 (15.8)	32 (84.2)	
Exposed to X-rated films in/ from childhood	Yes	27 (32.5)	56 (67.5)	^2^ = 0.151 p = 0.697
	No	64 (35)	119 (65.0)	
I take alcohol fairly regularly	Yes	19 (23.2)	63 (76.8)	^2^ = 6.419 p = 0.011*
	No	72 (39.1)	112 (60.9)	
I use drug/ other substances so as to feel high	Yes	11 (22)	39 (78)	^2^ = 4.079 p = 0.043*
	No	80 (37.0)	136 (63)	
I watch porn from time to time	Yes	9 (17.3)	43 (82.7)	^2^ = 8.205 p = 0.004*
	No	82 (38.3)	132 (61.7)	
Spends a lot of time online (internet usage)	Yes	49 (28.8)	121 (71.2)	^2^ = 6.073 p = 0.014*
	No	42 (43.8)	54 (56.3)	
Religiosity	Non-religious	12 (33.3)	24 (66.7)	^2^ =0.014 p = 0.905
	Religious	79 (34.3)	151 (65.7)	
Family Apgar	Severely dysfunctional	5 (45.5)	6 (54.5)	^2^ = 1.032# p = 0.589
	Moderately dysfunctional	23 (37.1)	39 (62.9)	
	Highly functional	63 (32.6)	130 (67.4)	

Over four fifths of respondents who were “not in terms with mum” and “those whose mum doesn't instruct morally” (86.7% and 84.2% respectively) were involved in RSB compared to 63.1% and 62.7% of those who were in good terms with mum and whose mum instruct them morally at significant value of p = 0.010 each. Two-thirds of the respondents who take alcohol fairly regularly (76.8%) were more involved in RSB compared to those that do not (60.9%) at p = 0.011. For the variables “Those that use drug/ other substance so as to feel high” (78%) and those that do not (63%); “those that watch porn from time to time” (82.7%) and those that do not watch porn (61.7%); and those that spend a lot of time online i.e. internet usage (71.2%) were involved in RSB compared to those that do not (56.3%) at p= 0.043, 0.004, 0.014 respectively as shown in Table 4.

Table 5 shows the binary logistic regression for the outcome variable “involvement in RSB and its possible predictors. None of the variables was statistically significant.

**Table 5:** Logistic regression for outcome variable “involvement in RSB” and its possible predictors

VARIABLES	SUB-VARIABLES	P VALUE	ODD'S RATIO	95% CONFIDENCE INTERVAL	
				Lower	Upper
Sex	Female	1			
	Male	0.178	1.530	0.823	2.844
Ever been treated of any STD	No	1			
	Yes	0.123	2.483	0.782	7.886
How many sexual partner have you ever had	Two or more partners	1			
	One	0.245	0.692	0.373	1.286
Good terms with mum	No	1			
	Yes	0.108	0.339	0.090	1.267
My mum instruct me morally	No	1			
	Yes	0.556	0.699	0.213	2.299
I take alcohol fairly regularly	No	1			
	Yes	0.512	1.269	0.622	2.588
I use drugs or other substance so as to feel high	No	1			
	Yes	0.665	1.215	0.503	2.934
Watch pornography	No	1			
	Yes	.300	1.616	.652	4.006
Spend more time online (internet usage)	No	1			
	Yes	0.286	1.407	0.752	2.632

## Discussion

Globally and locally, a number of research has been done on the prevalence and practices of RSB among youths /undergraduates but not much has been done on the factors / determinants of RSB. However, this study has identified some factors associated with risky sexual behavior among undergraduates.

Findings from this study showed that about seven out of ten respondents have had sex in the past 12 months /one year which is lower than the findings in another study^2^, which showed that about nine out of ten undergraduates has had sexual inter-course within last one year. The difference was probably due to large sample size of the previous study. The proportion of respondents who have had sex in the past one year was however slightly higher than that of a study(10) where 64.2% of in-school youths were self-reported to have had sexual intercourse in the past 12 months. This study showed more of males to be sexually experienced and active compared to their female counterparts which is similar to a previous study^10^. The apparent reason for this is that males are generally more comfortable talking or giving information about teir sexual escapades than females who might be more reserved. The other apparent implication of this finding is that more individuals, both males and females are likely to be having sex with multiple sexual partners concurrently since there is a significant disparity between the proportion of males and females having sex. This is based on the assumption that heterosexual intercourse is still the commonest sexual behavior/practice in this environment. The mean age at first sex in this study was 18.11 (±3.48) years and median was 18.01 which is comparable with previous study(10) which showed mean age of sexual initiation as 18.01 (±2.27) median age for female as 17.82 and males as 18.35 but different from another study which showed 16 years for females and 17 years for males^4^. The difference may probably be due to different age groups involved in the studies. Three fifths of the respondents had sex at the first time between ages 10–19, about a third had sex at first time at age 20–27 whereas 8.2% said they can't remember their age at first sex. Thus the majority of the respondents initiated sex early, this may not be unconnected with the environment in which they study as most are no longer with their parents and are often subject to peers, many of whom experiment with sex at their young ages.

A little over a quarter of the respondents have had more than one sexual partner concurrently, this is comparable to a study where 25% of respondents had more than one sexual partner at a time^10^. This study proceeded to know the total number of sexual partners the respondents have ever had, over a third have had only one sexual partner while almost two thirds have had two or more sexual partners in their lifetime. This is similar to another study where 62.3%^11^ and 63.4%^7^ of youths aged 20–24 years had multiple sexual partners (MSP). Some previous studies have reported lower proportion of respondents having MSP, for instance 54.6% has ever had MSP in an Ethiopia study^12^. The number of respondents involved with multiple sexual partners in this study is quite significant with a quarter currently having MSP and two thirds with a lifetime history of MSP, even at their young ages.

The main challenge with having MSP is the increased risk of acquiring STIs between such partners and STIS are a leading cause of morbidity and mortality among youths globally with respect to communicable diseases. With such high proportions of respondents having MSP, the likelihood that such a risky behavior will become more prevalent is very high as those currently involved in such relationships are likely to continue seeking other partners unless urgent educational interventions are carried out. Although the reported use of condom at last sexual intercourse in this study stood at just a little over half of the respondents, which was higher compared to some other findings^2^ where 85.8% reported not using condom in their last sexual activity but lower to another study where 65% reported using a condom at last sexual encounter^13^. The proportion of respondents using condoms at last sex is still dangerously low considering the risks involved. About a tenth of the respondents admitted to have been treated of any STI, out which those that do not use condom at last sexual intercourse and have already been treated of any STD was 51.6%. Thus half of those who have been previously treated for STIs still did not use condom at their last sex act. This is worrisome and may be a reflection of the often reported “care-free” attitude of youths when it comes to deriving sexual pleasures.

Consistent use of condom (100% of the time) among the sexually experienced and sexually active respondents was less than half in this study which is higher than another finding^12^ where 36.3% was reported but almost similar to that of a study on assessment of risky sexual behaviors and risk perception among youths in Western Ethiopia where 42.7% claimed consistent use of condom^10^. Inconsistent use of condom in itself is a principal risky sexual behavior as this study showed that 67.7% of those who were not consistent in condom usage have previously been treated of STIs. As stated earlier, it is an unhealthy development that majority of respondents previously treated for STIs are still inconsistent when it comes to condom use, perhaps it is a reflection of the fact that they have not come to terms with long-term effects of STIs compared to the transient pleasures of unprotected sex. Almost one-sixth of the respondents drank alcohol or used drugs/ stimulants at last sexual intercourse. Though this may seem as though it is a small proportion of respondents that were involved, in reality it is not, because such a risky behavior has a way of spreading among youths because of peer influence/ pressures. The use of alcohol and drugs before sex may be associated with disorientation and sexual violence with unprotected intercourse and consequent acquisition of STIs becoming increasingly prevalent.

When each of the three variables identified as risky sexual behaviours were computed for all respondents, almost two thirds of sexually experienced respondents were involved in at least one risky behaviour. In other words, two out of every three youths in this study were involved in RSB. This is quite high and reflects the significant level of risk being taken by youths when it comes to sex. In this study, some variables were found to have significant statistical association with respondents' involvement in RSB (p<0.005). These variables include sex (commoner in males), respondents who were not in good terms/relationship with mum, those whose mum doesn't instruct morally, respondents who took alcohol fairly regularly, use drugs/ other substance so as to feel high, watch pornography from time to time, spend a lot of time online i.e. internet usage and ever forcefully had sex with anyone. More male respondents were involved in RSB when compared with female and this is in tandem with previous study^14^,^15^. This development may be due to the explorative nature of males when it comes to risk-taking in general.

The respondents who were not in good relationship with mum and those whose mum did not instruct morally were identified to be involved in RSB in this study. This is in keeping with another research which showed that parental connectedness and monitoring are related to better sexual health as those with good parental connectedness and monitoring were less likely to engage in RSB than their counterparts^10^. This is possibly because parents have been identified as having a primary influence on young people's sexual behaviours and inadequate/ lack of parental supervision and education might well result in risky behaviours among youths and adolescent^14^. Mothers play an important role in determining/ modelling the behaviours their children engage in, most mothers spend quality time with their children compared to fathers. From infancy through childhood to early adulthood, not having good relationship with mum could possibly cut off the connectedness between young people and their parents and limit the parental supervision, education, moral instructions and/ or regulations of children's activities which may perhaps reduce the level of involvement in RSB and ultimately decrease the Sexual and Reproductive Health problems among youths. Youths who take alcohol faily regular, use drug/ other substance so as to feel high, watch porn from time to time and had ever forcefully had sex with someone were involved in RSB. Some of this characteristics have been found to be associated with RSB in other studies^2^,^16^–^20^.

## Limitation of study

The possible limitation of this study was that sensitive questions regarding respondents' sexual behaviours were asked and answers gotten via self-report. In this kind of situation, underreporting or embellishment on the part of respondents cannot be completely ruled out; however they were encouraged by the researchers to kindly give honest and accurate responses.

## Conclusion

This study has highlighted the prevalence of RSB and factors associated with it among undergraduates. The prevalence is high because as many as two out of three respondents were involved in RSB. Identified factors include: sex, having ever been treated of any STI, having ever had more than one sexual partner, having ever forcefully had sex with anyone, use of drugs so as to feel high, watching pornography, spending a lot of time online (internet usage), being in good terms with mum and receiving moral instructions from mother, which all showed statistical significance at p<0.05.

In other to curb the menace of RSB, among other important recommendations, parents especially mothers should be close enough to their children and make time out to give moral instructions to them as pertaining risky sexual behaviours and other related risks. This can be achieved by giving sexual health education at home to their children not only when they are undergraduates but right from childhood. Concerned stakeholders should enlighten the youths more on sexual health education as majority of them were involved in RSB, the use of behavioral change communication and intensified public health intervention strategies are recommended/ required to reduce the risky behaviours.

## Figures and Tables

**Figure 2:**
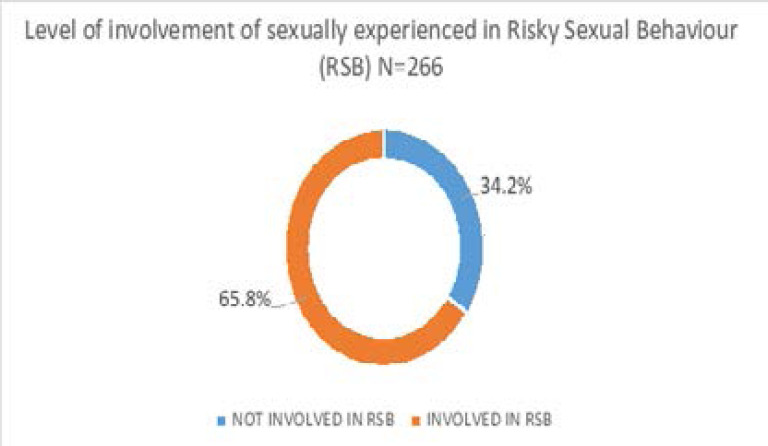
The figure above shows the proportion of respondents involved and uninvolved in risky sexual behavior which includes inconsistent use of condom, alcohol intake/ use of drugs for sexual activity and having multiple sexual partner concurrently, 65.8% (175) were involved in risky sexual behavior while 34.2% (91) were not involved in any risky sexual behavior.
